# Mixed Treatments Comparison of Oral Nutrition Interventions for Blood Immune Cell Parameters in Cancer Patients: Systematic Review and Network Meta-Analysis

**DOI:** 10.3390/metabo12090868

**Published:** 2022-09-15

**Authors:** Yufei Fang, Yining Xu, Yuting Zhang, Feng Ren, Julien S. Baker

**Affiliations:** 1 Hwa Mei Hospital, University of Chinese Academy of Sciences, Ningbo 315010, China; 2 Faculty of Sports Science, Ningbo University, Ningbo 315211, China; 3 Department of Sport and Physical Education, Hong Kong Baptist University, Hong Kong 999077, China

**Keywords:** nutrition, supplementation, immune, systematic review, network meta-analysis

## Abstract

Oral nutrition interventions are commonly applied as an assistant therapeutic approach, which could affect the balance of the immunological response but with mixed evidence. The objective of this study is to identify the potential of different oral nutrition interventions for blood immune cell parameters in cancer patients. Randomized controlled trials, which were published in peer-reviewed journals in the language of English, and which identified the effects of different oral nutrition interventions on cancer patients, were screened and included in the databases of PubMed, Medline, Embase, and Web of Science. White blood cell count (WBC), lymphocyte count, CD4/CD8, and neutrophil count were selected as outcome measures. For the result, 11 trials were included. The agreement between authors reached a kappa value of 0.78. Beta-carotene supplementation has a high potential in inducing a positive effect on blood immune cell parameters for cancer patients (first positive for WBC and CD4/CD8, second positive for lymphocyte count), as well as a combination of physical exercise and hypocaloric healthy eating intervention (first positive for lymphocyte and neutrophil count, second positive for WBC). Oral nutrition supplementations with a single substance have less potential to provide a positive effect on blood immune cell parameters for cancer patients (glutamine: 0.30 and 0.28 to be the last selection for WBCs and lymphocytes; Omega 3: 0.37 to be the last selection for WBCs; Protein: 0.44 to be the last selection for lymphocytes; Zinc: 0.60 to be the last selection for neutrophils). In conclusion, the programs of immunonutrition therapy for different cancer patients might be different. The past perception that mixed oral nutritional supplementations are superior to oral nutritional supplements with a single substance might be wrong and the selection of oral nutritional supplementation need cautiousness. A combination of physical exercise might have a positive effect but also needs a higher level of evidence. Registration Number: CRD42021286396.

## 1. Introduction

### 1.1. Rationale

Since one of the major characteristics of cancer is immune escape, monitoring the functioning of the immune system has a significant meaning during the treatments of cancers [1,2]. In recent years, immunonutrition has gradually become one of the hotspots in the related academic circle. Understanding the effect of nutrition and energy intake strategies on the functioning of the immune system in individuals with cancers has significant value in both the treatment and prevention processes of cancer. Under this situation, oral nutrition intervention is becoming one of the common assistant treatment protocols in cancer treatment; much evidence has identified that the nutrition intake, diet, and energy consumption would indirectly affect the body’s immune function of cancer patients because the metabolic processes could regulate immune cell responses [3,4,5,6]. For example, recent discoveries support a growing appreciation that microbial metabolites derived from bioactive foods are also important regulators of host immune and metabolic functions [7,8]. Moreover, a previous study has found that, in tumor-bearing mice, cyclic fasting or fasting-mimicking diets (FMDs) could enhance the activity of antineoplastic treatments by modulating systemic metabolism and boosting antitumor immunity and be safe, feasible, and resulting in a consistent decrease of blood glucose and growth factor concentration [9].

However, despite immunonutritional therapies seeming to have finally found their role in a wide range of tumors, several questions remain unanswered. Among these questions, the lack of validated biomarkers of response represents an important issue since only a proportion of cancer patients could benefit from immunotherapy. Based on these premises, a greater understanding of the role of potential biomarkers, including programmed death ligand 1 (PD-L1) expression, tumor mutational burden (TMB), microsatellite instability (MSI) status, gut microbiota, and several others, is necessary [10]. In addition, clinical trials on immunotherapy have widely differed in terms of drugs, patients, designs, terms of study phases, and inconsistent clinical outcomes [11].

When it comes to humans, as an important part of nutrition treatment protocols, oral nutrition interventions not only have a huge potential to provide a positive effect on the immune system function of cancer patients but also are very convenient in clinical practice. Oral nutrition interventions could be conducted at home so they are cheaper and easier to operate than enteral nutrition and injection, which could only be applied in hospitals and cost more money [12,13,14,15,16]. Some evidence has been provided to support the application of oral nutrition supplementation, but some have not. For example, the representative formula of Yanghe decoction in TCM was considered an important prophylactic and therapeutic treatment for breast cancer [17], which inhibits proliferation, reduces metastasis, and induces the apoptosis of breast cancer cells; its mechanism may be related to its inhibition of the activation of PI3K/Akt/NF-kB signaling pathway [18,19,20]. However, a study by Szefel’s team in the same year demonstrated that L-arginine supplementation did not support the hypothesis that L-arginine supplementation in colorectal cancer patients could reduce immunosuppression by decreasing the frequency of suppressor cells and increasing the frequency of effector CD4(+) T cells. It was not beneficial to the frequency of myeloid-derived suppressor cells and T lymphocytes in tumors and blood [21]. The potential mechanism of the heterogeneity might be that the integrated immune responses are correlated with dietary intake, energy utilization, and storage to immune regulation of tissue function [22]. At the same time, many trials have found paradoxical results of changes in nutrition and energy intake in the immune system function of both human and animal models [23,24].

At present, the best oral nutrition supplementation protocol for cancer patients is still unknown. The reasons were from many perspectives. Firstly, there are huge differences between different kinds of cancers [25]. Second, the function of the human immune system could be affected by many objective factors such as age, life habits, the gravity of the disease, comorbidities, etc. For example, the function of the human immune system will not change linearly with age; individuals with physical exercise habits may have a more frequent and lasting window of immune system function stress due to high-intensity exercise than sedentary ones [26,27]. Second, research published so far have mainly focusing on the correlation between nutrition and the functioning or state of the immune system in some special populations that need to pay attention to their immunometabolism, such as athletes [28], the elderly [29], infants [30], and pregnant females [31], as well as in individuals with metabolic or immune dysfunction such as type II diabetes, metabolic syndrome, or innate immunodeficiency [32,33,34]. Third, most of the present trials have focused on the correlations between different nutrition intake and diet strategies on the risk and mortality of cancer [35,36,37,38,39,40]. Last but not the least, the heterogeneity, which is created by the different designs and protocols of trials, results in vague and low-quality evidence for clinical practice.

The vagueness and heterogeneity of the evidence indicate the necessity of further comprehensive synthesis with a higher evidence level. According to the principle of evidence-based medicine (EBM), a registered systematic review with meta-analysis has the highest level in its evidence pyramid, and a network meta-analysis could compare more than two interventions synchronously, quantize and pool the effects of different treatment protocols together, and then rank these protocols according to a certain outcome measure. Therefore, a new systematic review with network meta-analysis is needed to make a mixed treatment comparison for different oral nutrition supplementation for cancer patients.

In addition, the results of the network meta-analysis provide the rank probabilities of interventions based on Bayes’ theorem and help clinical decision makers choose the optimal treatment protocols. Strictly speaking, the process of network meta-analysis is more in line with the spirit of EBM since its calculation is based on prior probabilities [41].

### 1.2. Objective

The objective of this systematic review is to identify the potential of different oral nutrition interventions for the blood immune cell parameters in cancer patients. It is the first network meta-analysis to identify the effects of different oral nutrition interventions on the blood immune cell parameters in cancer patients and could provide clinicians with high-level medical evidence for the control of immune indicators in the treatment of cancer patients. On the other hand, an adjusted and indirect comparison could compare more than two intervention protocols at the same time, bringing out information with more comprehensiveness for relevant clinical decisions.

## 2. Methods

### 2.1. Protocol and Registration

This systematic review was conducted according to the Preferred Reporting Items for Systematic Reviews and Meta-Analysis (PRISMA) extension statement for reporting systematic reviews incorporating network meta-analyses of health care intervention guidelines [42]. Literature eligibility and exclusion criteria and the search strategy were proposed and agreed on by two authors (Yufei Fang and Yuting Zhang) with a priori to minimize bias. The PROSPERO registration number of this review is CRD42021286396.

### 2.2. Eligibility Criteria

#### 2.2.1. Participants (P)

This systematic review included trials in which participants were patients (1) over 18 years old; (2) clinically diagnosed with non-digestive-tract cancer at all stages by oncologists; (3) clinically diagnosed with digestive tract cancer at all stages by oncologists; (4) without metastatic diseases.

#### 2.2.2. Interventions (I)

This systematic review included trials in which participants in experimental groups were provided oral nutritional supplementations or asked to have energy restriction eating strategies as interventions. All the included interventions were reclassified according to the following protocols: (1) Oral nutrition supplementations would be reclassified according to their nutrition substances, for example, an intervention in which patients were asked to take oral syrups of zinc sulfate at mealtimes was reclassified as “Zinc” group, whereas interventions in which patients were taking standard amino acids was reclassified as “Protein” group; (2) interventions in which patients were taking oral nutrition supplementations with more than one substance were reclassified as “Mixed” group; (3) interventions in which patients were asked to follow a plan that combined physical exercise and energy restriction were reclassified as “Lifestyle” group.

It needed to be emphasized that, since the absorption process of enteral nutrition treatments and injection treatments were different from that of the oral nutrition supplementation (as had been mentioned in the introduction), enteral nutrition treatments and injection treatments must be conducted at a hospital whereas oral nutrition supplementation could be conducted at home. For the consideration of minimizing the inconsistency and heterogeneities within trials, as well as the application in clinical practice, enteral nutrition treatments and injection treatments were excluded from this systematic review.

#### 2.2.3. Comparators (C)

This systematic review included trials in which participants in control groups were asked to maintain regular diets, conduct placebo intake protocols, or provide just patient education.

#### 2.2.4. Outcomes (O)

In clinical practice for cancer patients, blood immune cell parameters are the most used indicators to monitor the functioning of the immune system and the “Golden Standard” in the diagnosis of many immune-related diseases such as viral infection, inflammation, and stress response, and are also one of the important risk assessment factors in the process of cancer treatment. In the cancer treatment process, clinicians draw blood from their patients regularly, assess their blood immune cell parameters, and then choose the assistant treatment protocols or adjust the parameters in the treatment process such as the timing, type, or dose of drug administration according to the assessment results [43].

In this systematic review, white blood cell count (WBC), lymphocyte count, the ratio of CD4 and CD8 (CD4/CD8), and neutrophil count were selected as outcomes. Only studies with cancer patients whose blood immune cell parameters were in the normal range according to the clinical standard at the baseline were included. The normal range of blood immune cell parameters was: (1) WBC—from 4.0 × 10^9^/L to 10.0 × 10^9^/L; (2) lymphocyte count—from 800/mm^3^ to 4000/mm^3^; (3) CD4/CD8—from 1.4 to 2.0; (4) neutrophil count—from 1800/mm^3^ to 6300/mm^3^.

#### 2.2.5. Study Design (S)

Only randomized controlled trials were included in this systematic review.

#### 2.2.6. Exclusion Criteria

Trials were excluded if: (1) They applied non-oral nutrition interventions such as injections and enteral nutrition interventions; (2) participants were patients with different types of cancer, or the type of cancer was not specified; (3) the study was a published abstract without full text or lacked data; (4) outcome measures did not correspond with those in the eligibility criteria.

### 2.3. Information Sources

A comprehensive, reproducible search strategy had been performed on the databases of PubMed, Medline, Embase, and Web of Science from January 1990 to May 2022. Reference lists were also searched in all screened trials for identifying grey literature that might be potentially eligible. When the data of any eligible trials were insufficient, the authors were contacted and the missing data were requested.

### 2.4. Search

The search terms used in each database were as follows: (1) in PubMed and Embase, the search term was “((cancer) OR (tumor) [Titile/Abstract]) AND ((immun*) [Title/Abstract]) AND ((randomized) OR (randomised) [Title/Abstract])”; (2) in Medline and Web of Science, the search term was “(AB cancer OR tumor) AND (AB randomized OR randomised) AND (AB immun*) NOT (TI design or protocol or review)”. The search terms for eligible interventions were not limited in the database searching process since many terms could refer to oral nutrition supplementations.

### 2.5. Study Selection

The screening of the eligibility of intervention was conducted in the abstract and full-text screening process to guarantee that all the potentially eligible studies could be included in this systematic review.

Trials that were searched from the database were imported into EndNote 20 (Thomson Reuters, Carlsbad, CA, USA) to further screen and remove duplicates. Since there were no uniform keywords about oral nutrition interventions in the titles of trials searched from the databases, two independent authors (Yining Xu and Yuting Zhang) screened all the titles of the searched trials to identify all the potential trials before the abstract screening.

### 2.6. Data Collection Process

Data were extracted by two independent authors (Yufei Fang and Feng Ren).

### 2.7. Data Items

Details of trials were summarized and information such as population characteristics (age, gender, nationality, and type of cancer) and intervention protocols with their classification were collected and put into an extraction sheet which summarized the included trials. The data of each trial, which involved the sample size (N), mean value (Mean) with its standard deviation (SD) of each outcome of each group in baseline, and every data recording point, were recorded in an independent extraction sheet for the data preprocessing.

### 2.8. Geometry of the Network

The network geometry was made by the Aggregate Data Drug Information System (Version 1.16.8, http://drugis.org/software/addis/index, accessed on 1 July 2022) to display all kinds of interventions and key information, such as the type of intervention represented by each node, direct comparisons between each pair of interventions represented by the edges, and the arms of each comparison, which are represented by the number on every edge. Only interventions could be included in an adjusted indirect comparison, or a mixed treatment comparison would be analyzed in a network meta-analysis.

### 2.9. Risk of Bias within Individual Studies

The risk of bias within individual studies was assessed by two independent authors (Yining Xu and Yufei Fang) by applying the Cochrane Collaboration Risk of Bias Assessment Tool [44] in the Cochrane Library Review Manager software (Version 5.3, Wiley, Chichester, UK). An independent arbitrator (Ee-chon Teo) was invited when a disagreement occurred. The agreement between authors was represented by Cohen’s kappa value.

A study which had no items with high risk and which had less than 3 (contain) items with unclear risk were regarded as overall low risk; a study which had no item with high risk, but had more than 3 items with unclear risk, were regarded as an overall moderate risk; a study which had one item with high risk was also regarded as overall moderate risk, while a study which had more than one item with high risk was regarded as overall high risk.

### 2.10. Summary Measures

The effect size of the network meta-analysis was presented in the form of mean differences (MD).

The results under the consistency model were shown in the rank probability plot. The sum of all rank probabilities is 1, both within a rank over treatments and within a treatment over ranks. Moreover, a league table was provided after the model of data analysis had been determined, reporting results that represented the mean difference in the column-defining treatment compared with the row-defining treatment.

The results under the inconsistency model were shown in a league table [41].

### 2.11. Planned Methods of Analysis

Data preprocessing and analysis were conducted by two independent authors (Yining Xu and Feng Ren). Microsoft Office Excel (Version 16.0, Microsoft Corporation, Redmond, WA, USA) was used to preprocess the original data by transferring all the outcomes into a uniform unit according to the clinical criteria. In this review, the WBC data was transferred into the unit of 10^9^/L, the lymphocyte and neutrophil count were transferred into the unit of/mm^3^, and the CD4/CD8 was transferred into the standard decimal form that reserved two decimal fractions.

The Aggregate Data Drug Information System was used to pool data into the network meta-analysis and the Cochrane Library Review Manager (Version 5.3, Wiley, Chichester, UK) was applied to make the pair-wise meta-analysis.

In clinical practice, the medical nutrition treatment of most cancers aims to prevent the extreme increase of relevant immune cells induced by cancer and control the relevant immune cell count within the normal range. Therefore, in this review, the lower the blood immune cell parameters of WBC, lymphocyte, and neutrophil counts, the better. At the same time, CD4 mainly represents helper T cells and suppressor T cells, while CD8 represents killer T cells and cancer usually lowers CD4/CD8; therefore, in this review, the higher the CD4/CD8, the better.

### 2.12. Assessment of Inconsistency

The random-effects standard deviations were calculated under both consistency and inconsistency models and were compared with each other to identify if there was inconsistency within interventions. If there were closed loops in the intervention structure, the inconsistency of the evidence must be assessed. Moreover, while the results are easier to interpret, it requires a separate model to be run for each node to be split. The node-splitting analysis is an alternative method to assess inconsistency in network meta-analysis, which assesses whether direct and indirect evidence on a specific node (the split node) agree [45].

The consistency model was used if there was neither closed-loop nor split node in the intervention structure, the random-effects standard deviations in the consistency and inconsistency models were identical, or the identified discrepancy could be determined by examining the calculating a respective Bayesian *p*-value in the node-splitting analysis was statistically insignificant (*p* > 0.05). Otherwise, the inconsistency model should be applied [41].

### 2.13. Risk of Bias across Studies

The risk of bias across studies was assessed by two independent authors (Yining Xu and Yufei Fang) by applying the Cochrane Collaboration Risk of Bias Assessment Tool [44] in the Cochrane Library Review Manager software (Version 5.3, Wiley, Chichester, UK).

### 2.14. Additional Analyses

The Confidence in Network Meta-Analysis (CINeMA https://cinema.ispm.unibe.ch, assessed on 1 July 2022) was used to evaluate the confidence and assess the reporting bias in the findings from the network meta-analysis. According to the method research of CINeMA, if the item “within-study bias” was a “Major concern”, the confidence should be downgraded by one level. If other items were “Some concern”, the confidence would be downgraded by one level and if they were “Major concern”, the confidence would be downgraded by two levels [46,47].

The summarizing risk of bias assessments, which were set at “Average RoB”, applied a weighted average score for each relative effect estimate according to the percentage contribution of studies at each bias level. For example, studies of a direct comparison, which had low (arbitrarily assigned a score of 1), moderate (score 2), and high (score 3) risk of bias, had 40%, 25%, and 35% rate of contribution, and the total risk of bias score would be 0.40 × 1 + 0.25 × 2 + 0.35 × 3 = 1.95, which rounded to 2 and lead to “Some concerns” [46,47].

## 3. Results

### 3.1. Study Selection

**Figure 1:**
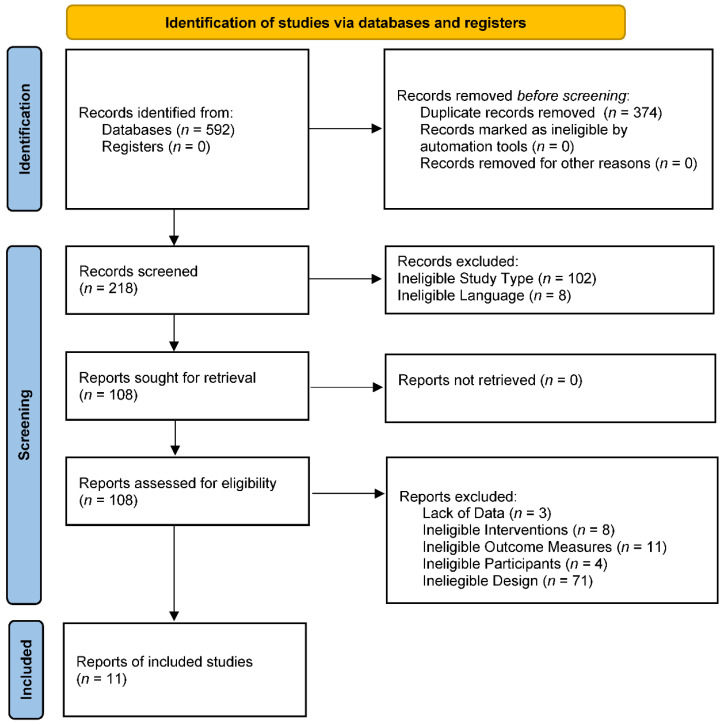
The PRISMA 2009 flow diagram of search and study selection.

Eleven trials were included in the final analysis [48,49,50,51,52,53,54,55,56,57,58]. The identification process was shown by a flow diagram, as in Figure 1.

There were nine categories of interventions included in this review, which were “Arginine”, “Beta-carotene”, “Glutamine”, “Omega 3”, “Protein”, “Zinc”, “Mixed”, “Lifestyle”, and “Control”. The information of all included trials is presented in Table 1. All the original data are provided in the Supplementary File.

### 3.2. Presentation of Network Structure

**Figure 2:**
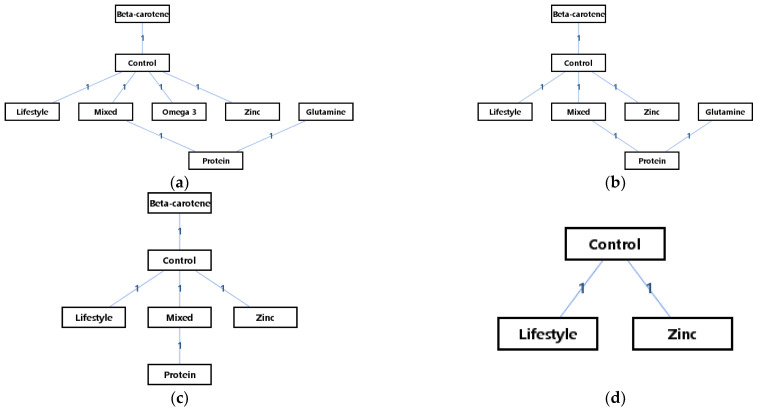
The network geometry of the interventions: (**a**) WBCs; (**b**) lymphocyte count; (**c**) CD4/CD8; (**d**) neutrophil count.

There were eight interventions in the network meta-analysis of WBC, seven interventions in the network meta-analysis of lymphocytes, four interventions in the network meta-analysis of CD4/CD8, and three interventions in the network meta-analysis of neutrophils. The network geometries displayed all kinds of treatments, providing key information such as the type of treatment represented by each node, the available direct comparisons between each pair of interventions (which is represented by the lines), and the arms of each trial (which are represented by the number on the edges). The network geometries of the interventions are presented in Figure 2. It can be seen that there was no closed loop in the outcome measures of the WBCs, lymphocyte count, CD4/CD8, and neutrophil count. Therefore, to determine whether to use the consistency or inconsistency model, what only needed to be conducted was the comparison of the random-effects standard deviation in each result of outcome measures.

### 3.3. Study Characteristics

Characteristics of included studies are provided in Table 1.

### 3.4. Risk of Bias within Studies

**Figure 3:**
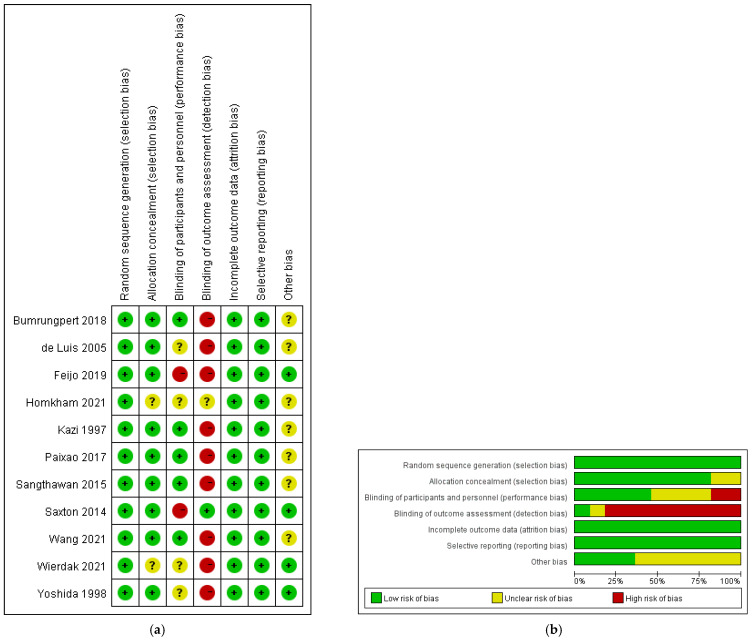
The result of the risk of bias assessment. (**a**) Risk of bias summary [48,49,50,51,52,53,54,55,56,57,58]; (**b**) risk of bias graph.

A consensus was reached for all items with a kappa value of 0.78. The results of the risk of bias assessment are shown in Figure 3. It can be seen that two trials had a high risk of bias, seven trials had a moderate risk of bias, and two trials had a low risk of bias. The risk of performance bias (blinding of participants and personnel) was moderate (high in two trials and unclear in four trials); (2) the risk of detection bias (blinding of outcome assessors) was high (high in nine trials and unclear in one trial); (3) the risk of attrition bias (incomplete outcome data) was low (low in all trials); (4) the risk of selection bias (random sequence generation and allocation concealment) was low (low in all trials); (5) the risk of reporting bias (selective reporting of outcomes) was low (low in all trials).

### 3.5. Results of Individual Studies

The results of individual studies are summarized and provided in Table 2.

### 3.6. Synthesis of Results

**Figure 4:**
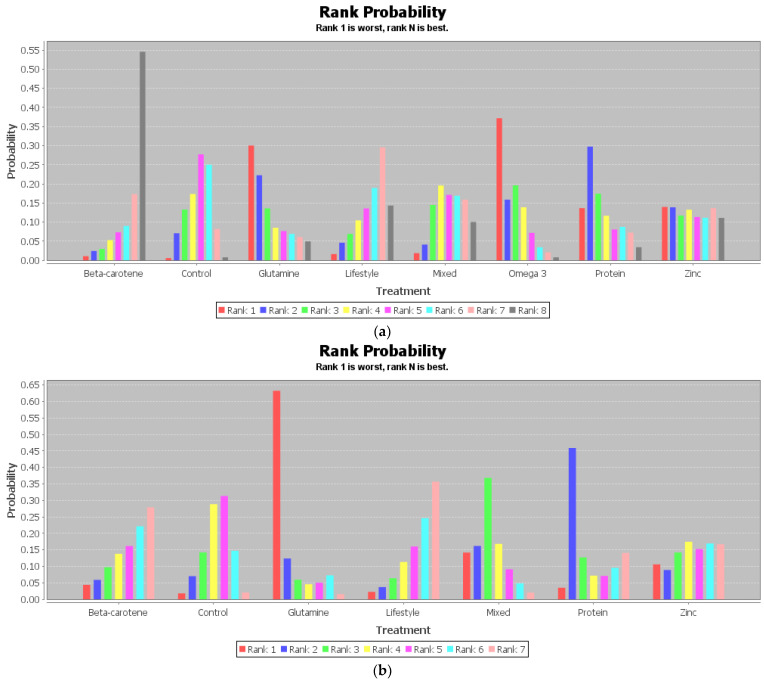

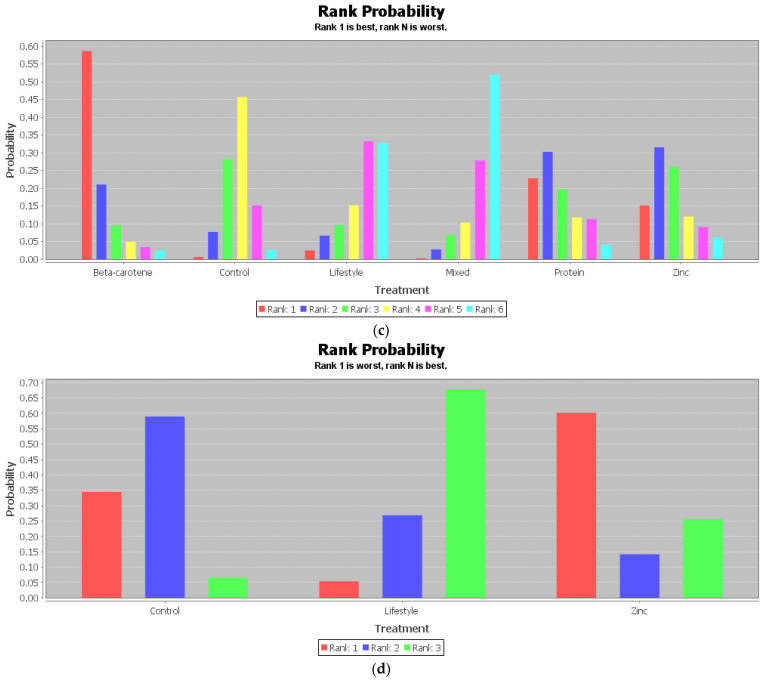
Ranking of measures and probabilities: (**a**) WBCs; (**b**) lymphocyte count; (**c**) CD4/CD8; (**d**) neutrophil count.

Table 3 is the league table of the network geometries, and the ranking of measures and probabilities are provided in Table 4 and Figure 4. What should be paid attention to is that in the probabilities ranking figure of CD4/CD8, as in Figure 4c, the rank N was the worst one, and rank 1 was the best one, whereas in those of WBC, lymphocyte, and neutrophil, the rank N was the best one, and rank 1 was the worst one.

It can be seen that beta-carotene supplementation had a 0.55 probability to be the best intervention for WBCs, a 0.59 probability to be the best intervention for CD4/CD8, and a 0.27 probability to be the sub-best intervention for lymphocytes. Changing lifestyle, which referred to a daily calorie intake of 600 kcal below the energy requirements with additional supervised exercise sessions, had a 0.36 probability to be the best intervention for lymphocytes and a 0.68 probability to be the best intervention for the neutrophil count.

### 3.7. Explanation for Inconsistency

The results of the random-effects standard deviation calculations in both the consistency model and inconsistency model of each outcome measure are provided in Table 5 in the form of the mean value and its 95% confidence intervals. According to the results, the random-effects standard deviations of the consistency model and that of the inconsistency model in the network structure of each outcome measure were well identical (*p* > 0.05). It means that the analysis under the consistency model had good validity.

### 3.8. Results of Additional Analyses

Table 6 provides the results of the confidence assessment made by CINeMA. According to Table 6, except for the mixed comparison of glutamine and protein, and the indirect comparisons of beta-carotene and glutamine, in terms of the effect on leukocytes (WBC) in blood for cancer patients, control treatment and glutamine, control treatment and protein, glutamine and lifestyle change, glutamine and mixed supplementation protocol, glutamine and omega 3, and glutamine and zinc all had low confidence ratings; all other indirect and mixed evidences had a very low confidence rating.

## 4. Discussion

### 4.1. Summary of Evidence

The objective of this systematic review was to identify the potential of different oral nutrition interventions for the blood immune cell parameters in cancer patients. The main findings are as follows. First, according to the results of the network meta-analysis, for cancer patients and without considering the effect size, intaking beta-carotene seems to be the supplementation protocol with the most potential for inducing a positive effect on the blood immune cell parameters, whereas the change in lifestyle—which was a low daily calorie intake with additional supervised exercise sessions—seems to be another potential protocol to induce a positive effect on the blood immune cell parameters. Second, other oral nutrition supplementation protocols, such as glutamine, protein (or amino acids), zinc, and mixed substance, seemed not effective as hoped. Third, although the effect sizes were statistically insignificant from very small to small, the overall results of the pair-wise meta-analysis supported the advantage of oral nutrition interventions over the interventions used in control groups, which were regular diets, placebo intake protocols, and patient education. Last but not the least, different nationalities of patients might affect the effect of oral nutrition intervention on blood immune cell parameters.

Part of these results corresponds with the demonstration of some previous trials. First, according to the results of the pair-wise meta-analysis, giving oral nutrition supplementations had a statistically insignificant small but positive effect on the blood immune cell parameters for patients with cancer. This finding corresponded with the results of a systematic review that assessed the effects of glutamine, arginine, and omega-3 supplementation on the tolerance to treatment, nutritional status, and immune function of head and neck cancer patients undergoing chemoradiotherapy, claiming that the glutamine supplementation could significantly reduce the risk of mucositis [59]. Second, the results of the network meta-analysis of this systematic review identified the high potential of beta-carotene supplementation protocols in preventing the immune cell parameters from extremely increasing (ranked first positive for WBC and CD4/CD8, second positive for lymphocyte count). Some previous trials presented similar results to that of this systematic review. For instance, a study published in 2016 claimed that beta-carotene might have an immune-enhancing effect through the production of Th1 cytokines by activation of splenocytes and macrophages [60]; a study whose participants were workers engaged in the copper-smelting industry found that preventive use of beta-carotene could prevent negative changes in immunological parameters for the participants [61]; and a randomized, double-blind controlled trial in 2010 showed that maternal supplementation including beta-carotene would affect the newborn’s immune development in specific ways [62]. Third, another important finding of this review is the large potential of the combination of physical exercise and hypocaloric healthy eating, which was allocated in the classification of “Lifestyle”, to provide a positive effect on the immune cell parameters for cancer patients. Some previous trials have verified the positive effect of energy restriction strategies on the functioning of the human immune system; a study of an animal model conducted in 2004 demonstrated that energy restriction could restore the impaired immune response in overweight rats [23], and another animal trial in 1994 found that energy restriction could prevent and reverse immune thrombocytopenic purpura and increases the life span of mice [63]. An important human study published in 1998 claimed that energy restriction was associated with a significant decrease in mitogen-stimulated lymphocyte proliferation, but no change in natural killer cell activity, monocyte and granulocyte phagocytosis and oxidative burst, or symptoms of upper respiratory tract infection [24]. A narrative review published in 2008 supported the role of physical exercise and energy restriction in the treatment process of cancer, demonstrating that some key biological mechanisms were providing important metabolic links between nutrition, physical activity, and cancer, including insulin resistance and reduced glucose tolerance, increased activation of the growth hormone/IGF-I axis, alterations in sex-steroid synthesis and/or bioavailability, and low-grade chronic inflammation through the effects of adipokines and cytokines [64]. Last, the pair-wise meta-analysis found that oral nutrition interventions had a small and insignificant advantage over regular diets, placebo intake protocols, and patient education with the heterogeneities potentially coming from patients’ nationalities or differences in treatment protocols [65,66,67,68].

However, some previous trials and reviews hold different viewpoints. For example, a study published in 2014 claimed that the evidence to recommend routine use of immune nutrition in patients undergoing esophageal cancer surgery was still insufficient [69], and a review published in 2014 declared that there was not enough evidence in malnourished urological study cohorts to establish a consensus on immune-nutrition and the role of immune-nutrition should be considered investigational in patients with bladder cancer until there are more well-controlled comparative effective trials or randomized trials [70]. Another systematic review published in 2006 that included randomized controlled trials examined the effects of nutritional interventions on patients with cancer or preinvasive lesions and demonstrated that there was no evidence that dietary modification by cancer patients could improve survival and benefit disease prognosis because of the limited number of high-quality trials [71]. Moreover, the evidence to support applying beta-carotene in cancer patients is still weak. A randomized controlled trial of Dunstan’s team identified that supplementation with beta-carotene did not affect the antioxidant status and immune responses in allergic adults [72], and a randomized prospective study conducted in 2000 found that beta-carotene could only enhance the cytotoxicity of NK cells but could not affect phenotypic expression of T cell subsets [73]. Moreover, one should be careful to interpret this result since the causal relationship between the energy restriction and the improvement of blood immune cell parameters is still unclear. On one hand, malnutrition is commonly reported in cancer patients. On the other hand, there are various ways to create energy deficiency and many different energetic balance equation hypotheses. Therefore, caution should be paid when planning to apply the energy restriction strategies in the process of oral nutrition treatment for cancer patients. Additionally, the evidence from this perspective is also vague since some other previous trials demonstrated the positive effect provided by certain oral nutrition supplements. An animal study finished in 2021 claimed that dietary palmitic acid could promote metastasis in oral carcinomas and melanoma in mice; tumors from mice that were fed a short-term palm-oil-rich diet, or tumor cells that were briefly exposed to PA in vitro, remained highly metastatic even after being serially transplanted [74]. A systematic review and meta-analysis suggest that parenteral omega-3 fatty acid supplementation was beneficial for gastrointestinal cancer patients, and was accompanied by improved postoperative immune function and satisfactory clinical outcomes [75].

What should be paid more attention is that the controversy surrounding beta-carotene is not limited to its effect on the function of the immune system. As has been mentioned in the introduction, clinical practice is more concerned with the safety indicators, such as mortality and morbidity, when it comes to cancer treatments. However, the results of some previous studies have raised concerns about the safety of beta-carotene for cancer patients. For example, a randomized trial conducted by Bairati’s team in 2006 found increased mortality in head and neck cancer patients who were supplemented with alpha-tocopherol and beta-carotene [76]. Similar results were reported in a systematic review with a broader population included as participants. A Cochrane systematic review published in 2012 assessed the beneficial and harmful effects of antioxidant supplements for the prevention of mortality in 269,707 adults, claiming that eta-carotene seemed to increase mortality and should be considered as medicinal products and should undergo sufficient evaluation before marketing [77]. Moreover, Bjelakovic’s team examined the association between beta-carotene and mortality based on their 2012 Cochrane systematic review to assess whether different doses of beta-carotene affected mortality in primary and secondary prevention randomized clinical trials with low risk of bias by using meta-analyses, meta-regression, and trial sequential analyses. Eventually, Bjelakovic’s team concluded that beta-carotene in doses higher than the recommended daily allowances seemed to significantly increase mortality [78]. Considering all the information above, beta-carotene supplementation has the potential to improve the immunometabolism of cancer patients, but their chances of mortality might be significantly higher. The heterogeneity between trials might come from their different intervention protocols and different populations of participants. For example, the detailed physiological mechanics of beta-carotene’s functioning in the human body is still lacking exploration. A cross-sectional study published in 2000 claimed that plasma beta-carotene lacked association with the immune response to the influenza vaccine in the healthy elderly [79]; however, a double-blind, placebo-controlled, crossover study, whose subjects were adult male nonsmokers, found that after dietary supplementation of beta-carotene, there were significant increases in plasma levels of beta-carotene and the percentages of monocytes expressing the major histocompatibility complex class II molecule HLA-DR, the adhesion molecules intercellular adhesion molecule-1, the leukocyte function-associated antigen-3, and the ex vivo TNF-alpha secretion by blood monocytes were significantly increased [80]. Considering that all the trials related to the beta-carotene supplementation included in this review were single-arm and their results were not statistically significant, more high-quality research is needed in the future to clarify the effects of beta-carotene, explain its mechanism, and provide the best guideline by comparing different intake protocols. When it comes to the patient population, the results of subgroup analysis in the pair-wise meta-analysis identified the potential that the nationalities might become one of the heterogeneity sources since the I2s between subgroups were larger than for those within the overall effects. The heterogeneity that came from the nationalities also indicated the possibility of publication bias, which was mainly induced by the lack of trials with participants from East Asia.

To sum up, oral nutrition intervention for cancer patients is a complex issue. Although this review and other previous trials failed to verify a significant positive effect of any oral nutrition supplementation protocol on immune cell parameters of cancer patients, the result of this review could still provide important enlightenment for future research because of two main strengths. On one hand, the network meta-analysis based on the Bayesian approach indicated that the preconception that oral nutrition supplementation must have a positive effect on cancer patients should be avoided and the importance of lifestyle interventions and overall energy intake control could not be neglected. On the other hand, the results of subgroup analysis and publication bias assessment in the pair-wise meta-analysis indicated that further trials should focus on the comparison of cancer patients of different races or nationalities to identify the different effects of oral nutrition interventions.

### 4.2. Limitations

First, the meta-analysis in this review did not include clinical outcomes such as mortality, morbidity, or adverse events such as malnutrition and immunological stress reaction [81,82,83]. It could not be ignored that the surrogate outcome measures, especially laboratory indices—which were very often unreliable substitutes in clinical practice—were applied instead of clinical outcomes, inducing potential dangers when assessing new treatment protocols. The ideal primary outcomes should be relevant to the patient’s quality of life or the course of the disease; a significant correlation between a surrogate and a clinical outcome could not explicitly mean that the observed beneficial effect of an intervention on the surrogate outcome will be the same on the clinical outcome.

Second, since the monitoring of blood immune cell parameters in cancer treatment is usually continuous, the outcome measured at baseline and at each endpoint could only represent the current status [75]. Unfortunately, since there were only a few studies included in these comparisons, the publication bias in the comparisons of some outcome measures, such as CD4/CD8 and the neutrophil count, could not be quantitatively evaluated, and the confidence of evidence for CD4/CD8 and the neutrophil count is also unknown.

Third, there was a lot of variation between the studies in terms of gender, tumor type, treatment, and stage of the disease, and there was very small number of studies representing a given type of intervention. Moreover, the different tumor types would affect the outcomes and results. For example, digestive tract cancers, such as colon cancer, which could block the absorption of nutrition, may derive maximum immunonutrition support from enteral nutrition regimens [84,85,86].

Last, the race and nationality of the participants were not limited in the eligibility criteria of the participants. However, different races differ in the risk of different types of cancer, nutritional needs, and dietary habits. There is a lack of relevant high-quality evidence.

### 4.3. Conclusions

According to the change in blood immune cell parameters, it could be inferred that the programs of immunonutrition therapy for different cancer patients might be different. Moreover, the past perception that mixed oral nutritional supplementations are superior to oral nutritional supplements with a single substance might be wrong, at least from the mathematical perspective in this review. Therefore, the selection of oral nutritional supplementation needs cautiousness. Finally, a combination of physical exercise might have a positive effect on the immune function of cancer patients, and more relevant high-quality studies should be conducted in the future.

## Figures and Tables

**Table 1:** Study Characteristics.

Study	Participants					Interventions			Outcome Measures
	Age	Gender (F/M)	Cancer	Nationality	Protocol	Process	Classification	Therapy Form	
Kazi 1997 [54]	67.47	3/16	Colon cancer	American	Beta-carotene capsules	30 mg/day, 3 months	Beta-carotene	After surgery	WBC Lymphocyte CD4/CD8
					Placebo capsules	30 mg/day, 3 months	Control		
Yoshida 1998 [51]	61.18	2/11	Esophageal cancer	Japanese	Oral glutamine	30 g/day, 28 days,	Glutamine	Irradiation and chemotherapy	WBC Lymphocyte
					Standard amino acid solution	Isonitrogenous aminos, 28 days	Protein		
de Luis 2005 [56]	61.8	5/68	Head and Neck cancer	Spanish	Omega 3-enhanced oral immunonutrition	Omega 3-enhanced supplementation with a basal oral diet, 12 weeks	Omega 3	After surgery	Lymphocyte
					Arginine-enhanced oral immunonutrition	Arginine-enhanced supplementation with a basal oral diet, 12 weeks	Arginine		
Saxton 2014 [49]	55.56	85/0	Early-stage breast cancer	British	An exercise and hypocaloric healthy eating intervention	600 kcal below their calculated energy requirements/day + 3 supervised exercise sessions (30 min aerobic exercise + 10 to 15 min of muscle-strengthening exercises)/week	Lifestyle	After surgery	WBC Lymphocyte CD4/CD8 Neutrophil
					Blank	A healthy eating booklet	Control		
Sangthawan 2015 [53]	61.00	8/64	Head and Neck cancer	Thai	Zinc sulfate supplementation	Oral syrups zinc sulfate, 5 mg/cc, 50 mg (10 cc)/meal, 3 times/day at mealtimes	Zinc	Radiation Therapy after surgery	WBC Lymphocyte CD4/CD8 Neutrophil
					Placebo	Oral syrups of a placebo, 3 times/day at mealtimes	Control		
Paixao 2017 [50]	51.06	37/0	Breast cancer	Brazilians	EPA and DHA-enriched fish oil	2 g/day of fish oil concentrate containing 1.8 g of n-3 fatty acids for 30 days	Omega-3	Perioperative period	WBC
					Placebo	2 g/day of mineral oil for 30 days	Control		
Feijo 2019 [52]	58.00	22/44	Gastric cancer	Brazilians	Omega-3 supplementation	600 kcal, 24 g protein, and 3.2 g of omega 3/day, 200 mL/day, 30 days	Mixed	Before Surgery	CD4/CD8
					Standard formula without Omega-3	560 kcal and 29 g protein/day, 30 days	Protein		
Wierdak 2021 [55]	64.26	14/12	Colorectal cancer	Polish	Standard oral nutritional	2 times Nutricia Nutridrink Protein/day, 2/day, 2 weeks	Protein	Perioperative period	WBC Lymphocyte Neutrophil
					Immunonutrition	2 times Arginine + Glutamine + Omega-3 + Nucleotides + Zinc, 2 weeks	Mixed		
Wang 2021 [58]	55.4	36/0	Breast cancer	Chinese	Spleen amino-peptide oral lyophilized powder	4 mg on the first day of chemotherapy for two cycles.	Mixed	Unlimited	CD4/CD8
					Placebo	4 mg on the first day of chemotherapy for two cycles.	Control		
Bumrungpert 2018 [57]	52.92	32/10	Cancer without metastatic diseases	Thai	Whey Protein Supplementation	40 g Whey protein isolate with Zn (2.64 mg/day) and Se (0.76 mg/day)	Mixed	During chemotherapy	WBC
					Maltodextrin oral snack	40 g of maltodextrin as a daytime snack	Control		
Homkham 2021 [48]	56.00	35/49	Cancer	Thai	Regular diet	1500 kcal, 60 g protein/day (esophageal cancer patients, 2000 kcal, 75 g/day via feeding tube)	Control	During chemo-therapy	Lymphocyte
					Immune-enhanced nutritional supplementation	A regular diet + 500 kcal/day of supplementation containing arginine 6.16 g, L-glutamine 3.07 g, and fish oil 2.73 g that prepared in sachet form, 2 times/day	Mixed		

WBC: white blood cell count (Leukocytes).

**Table 2:** Results of Individual Studies.

Study	Duration	Reporting Time	Main Results of Blood Immune Cell Parameters
Kazi 1997 [54]	12 weeks	Pre treatment 12 weeks	A significant increase in lymphocytes and CD4.
Yoshida 1998 [51]	4 weeks	Pre treatment 4 weeks	A reduction in the lymphocyte count; A blast formation of lymphocytes and the amount of phenolsulfonphthalein excretion in the urine was greater in the control than in the glutamine group.
de Luis 2005 [56]	12 weeks	Pre treatment 12 weeks	No significant intergroup differences in the trend of the three serum proteins and lymphocytes were detected.
Saxton 2014 [49]	24 weeks	Pre treatment 24 weeks	Women in the control group had higher total leukocyte, neutrophil, and lymphocyte counts in comparison to the intervention group at the 6-month follow-up.
Sangthawan 2015 [53]	Unlimited	Pre treatment 5 weeks Post treatment	White blood cell and neutrophil counts all continuously decreased from baseline to 1-month follow-up and again no significant differences in the 2 groups were detected; The pattern of responses of circulating total lymphocytes, total T lymphocytes (CD3), and T lymphocyte subpopulations (CD4 and CD8) were similar in the 2 groups; The absolute numbers of T lymphocyte parameters of both groups continued to decrease after starting radiation therapy to lower than 50% of the baseline level at week 5 of radiation therapy and increased slightly in the first month after completion of radiation therapy; There were no statistically significant differences between the 2 groups at any of the 3 time points.
Paixao 2017 [50]	30 days	Pre treatment 30 days	The percentages of peripheral blood CD4(+) T lymphocytes and serum high sensitivity C-reactive protein (hsCRP) levels were maintained in the control group; A significant reduction in the percentage of CD4(+) T lymphocytes in the peripheral blood in the experimental group.
Feijo 2019 [52]	30 days	Pre treatment 30 days	There was the maintenance of the immune profile in both groups;
Wierdak 2021 [55]	2 weeks	Pre treatment 2 weeks	In both groups, a decrease in superficial neutrophil infiltration was observed, but this was only statistically significant in the immune group;
Wang 2021 [58]	12 weeks	Pre treatment 3 weeks 6 weeks 12 weeks	On day 84, the number of CD3, CD4, and CD8 cells was significantly higher in the experimental group;
Bumrungpert 2018 [57]	12 weeks	Pre treatment 6 weeks 12 weeks	Whey protein supplementation significantly increased albumin and immunoglobulin G levels compared to the control group at week 12; There was a significant time-dependent increase in the intervention group.
Homkham 2021 [48]	4 weeks	Pre treatment 2 weeks 4 weeks	Neutrophil to lymphocyte ratio and absolute lymphocyte count at baseline was significantly associated with dynamic changes in neutrophil to lymphocyte ratio and absolute lymphocyte count; The magnitudes of the neutrophil to lymphocyte ratio and absolute lymphocyte count changes through treatment were lower than in control; The differences were not statistically significant except for absolute lymphocyte count at the end of treatment.

**Table 3:** The league tables of the network geometries.

**WBC**	**Beta-carotene**	1.26	2.23	0.81	1.20	2.37	2.04	1.45
		**Control**	0.99	−0.44	−0.06	1.10	0.84	0.22
			**Glutamine**	−1.42	−1.05	0.13	−0.16	−0.76
				**Lifestyle**	0.37	1.55	1.25	0.64
					**Mixed**	1.16	0.88	0.25
						**Omega 3**	−0.28	−0.89
							**Protein**	−0.63
								**Zinc**
**Lymphocyte**	**Beta-carotene**	58.54	318.67	−29.41	149.02	186.55	57.62	
		**Control**	260.49	−87.11	89.32	129.54	−1.97	
			**Glutamine**	−344.58	−171.71	−132.38	−263.43	
				**Lifestyle**	176.71	215.26	84.98	
					**Mixed**	39.20	−89.82	
						**Protein**	−132.57	
							**Zinc**	
**CD4/CD8**	**Beta-carotene**	−0.62	−0.84	−0.96	−0.35	−0.40		
		**Control**	−0.22	−0.33	0.26	0.22		
			**Lifestyle**	−0.11	0.49	0.44		
				**Mixed**	0.60	0.55		
					**Protein**	−0.04		
						**Zinc**		
**Neutrophil**	**Control**	−376.33	285.34					
		**Lifestyle**	650.94					
			**Zinc**					

WBC: white blood cell count (Leukocytes); Lifestyle: a combination of physical exercise and hypocaloric healthy eating.

**Table 4:** Ranking of measures and probabilities.

Outcome	Intervention	Rank 1	Rank 2	Rank 3	Rank 4	Rank 5	Rank 6	Rank 7	Rank 8
WBC	Beta-carotene	0.01	0.02	0.03	0.05	0.07	0.09	0.17	0.55
	Control	0.01	0.07	0.13	0.17	0.28	0.25	0.08	0.01
	Glutamine	0.30	0.22	0.14	0.09	0.08	0.07	0.06	0.05
	Lifestyle	0.02	0.05	0.07	0.10	0.14	0.19	0.30	0.14
	Mixed	0.02	0.04	0.14	0.20	0.17	0.17	0.16	0.1
	Omega 3	0.37	0.16	0.20	0.14	0.07	0.03	0.02	0.01
	Protein	0.14	0.30	0.17	0.12	0.08	0.09	0.07	0.03
	Zinc	0.14	0.14	0.12	0.13	0.11	0.11	0.14	0.11
Lymphocyte	Beta-carotene	0.04	0.06	0.10	0.13	0.16	0.24	0.27	
	Control	0.00	0.03	0.14	0.32	0.35	0.14	0.02	
	Glutamine	0.28	0.32	0.22	0.11	0.04	0.02	0.01	
	Lifestyle	0.02	0.03	0.06	0.10	0.15	0.28	0.36	
	Mixed	0.13	0.30	0.26	0.14	0.09	0.05	0.02	
	Protein	0.44	0.11	0.08	0.06	0.07	0.08	0.16	
	Zinc	0.09	0.13	0.14	0.14	0.15	0.18	0.16	
CD4/CD8	Beta-carotene	0.59	0.21	0.1	0.05	0.03	0.02		
	Control	0.01	0.08	0.28	0.46	0.15	0.03		
	Lifestyle	0.02	0.07	0.1	0.15	0.33	0.33		
	Mixed	0	0.03	0.07	0.1	0.28	0.52		
	Protein	0.23	0.3	0.2	0.12	0.11	0.04		
	Zinc	0.15	0.32	0.26	0.12	0.09	0.06		
Neutrophil	Control	0.34	0.59	0.07					
	Lifestyle	0.05	0.27	0.68					
	Zinc	0.60	0.14	0.26					

WBC: white blood cell count (Leukocytes); Lifestyle: a combination of physical exercise and hypocaloric healthy eating.

**Table 5:** The results of the random-effects standard deviation calculations.

Outcome	Model	Inference Samples	Random-Effects Standard Deviation	*t*	Sig.
**WBC**	**Consistency**	10,000	0.64 (0.06, 1.23)	0.042	0.97
	**Inconsistency**	20,000	0.62 (0.04, 1.23)		
**Lymphocyte**	**Consistency**	40,000	67.09 (5.76, 127.80)	0.001	0.99
	**Inconsistency**	40,000	64.07 (3.34, 127.67)		
**CD4/CD8**	**Consistency**	10,000	0.32 (0.02, 0.62)	0.031	0.98
	**Inconsistency**	40,000	0.33 (0.02, 0.63)		
**Neutrophil**	**Consistency**	10,000	193.51 (12.39, 369.40)	0.017	0.99
	**Inconsistency**	20,000	191.02 (10.13, 369.41)		

WBC: white blood cell count (Leukocytes).

**Table 6:** Results of the confidence rating.

Outcome	Structure	Comparison	Arms	Within-Study Bias	Reporting Bias	Indirectness	Imprecision	Heterogeneity	Incoherence	Confidence Rating	Reason(s) for Downgrading
**Leukocytes (WBC)**	**Mixed**	**Beta-carotene:Control**	1	Some concerns	Low risk	No concerns	Major concerns	No concerns	Major concerns	Very low	Imprecision and Incoherence
		**Control:Lifestyle**	1	Some concerns	Low risk	No concerns	Major concerns	No concerns	Major concerns	Very low	Imprecision and Incoherence
		**Control:Mixed**	2	Some concerns	Low risk	No concerns	Major concerns	No concerns	Major concerns	Very low	Imprecision and Incoherence
		**Control:Omega 3**	1	Some concerns	Low risk	No concerns	Major concerns	No concerns	Major concerns	Very low	Imprecision and Incoherence
		**Control:Zinc**	2	Some concerns	Low risk	No concerns	Major concerns	No concerns	Major concerns	Very low	Imprecision and Incoherence
		**Glutamine:Protein**	3	Some concerns	Low risk	No concerns	No concerns	No concerns	Major concerns	Low	Incoherence
		**Mixed:Protein**	1	Some concerns	Low risk	No concerns	Major concerns	No concerns	Major concerns	Very low	Imprecision and Incoherence
	**Indirect**	**Beta-carotene:Glutamine**	0	Some concerns	Low risk	No concerns	No concerns	No concerns	Major concerns	Low	Incoherence
		**Beta-carotene:Lifestyle**	0	Some concerns	Low risk	No concerns	Major concerns	No concerns	Major concerns	Very low	Imprecision and Incoherence
		**Beta-carotene:Mixed**	0	Some concerns	Low risk	No concerns	Major concerns	No concerns	Major concerns	Very low	Imprecision and Incoherence
		**Beta-carotene:Omega 3**	0	Some concerns	Low risk	No concerns	Major concerns	No concerns	Major concerns	Very low	Imprecision and Incoherence
		**Beta-carotene:Protein**	0	Some concerns	Low risk	No concerns	Major concerns	No concerns	Major concerns	Very low	Imprecision and Incoherence
		**Beta-carotene:Zinc**	0	Some concerns	Low risk	No concerns	Major concerns	No concerns	Major concerns	Very low	Imprecision and Incoherence
		**Control:Glutamine**	0	Some concerns	Low risk	No concerns	No concerns	No concerns	Major concerns	Low	Incoherence
		**Control:Protein**	0	Some concerns	Low risk	No concerns	Major concerns	No concerns	Major concerns	Very low	Imprecision and Incoherence
		**Glutamine:Lifestyle**	0	Some concerns	Low risk	No concerns	No concerns	No concerns	Major concerns	Low	Incoherence
		**Glutamine:Mixed**	0	Some concerns	Low risk	No concerns	No concerns	No concerns	Major concerns	Low	Incoherence
		**Glutamine:Omega 3**	0	Some concerns	Low risk	No concerns	No concerns	No concerns	Major concerns	Low	Incoherence
		**Glutamine:Zinc**	0	Some concerns	Low risk	No concerns	No concerns	No concerns	Major concerns	Low	Incoherence
		**Lifestyle:Mixed**	0	Some concerns	Low risk	No concerns	Major concerns	No concerns	Major concerns	Very low	Imprecision and Incoherence
		**Lifestyle:Omega 3**	0	Some concerns	Low risk	No concerns	Major concerns	No concerns	Major concerns	Very low	Imprecision and Incoherence
		**Lifestyle:Protein**	0	Some concerns	Low risk	No concerns	Major concerns	No concerns	Major concerns	Very low	Imprecision and Incoherence
		**Lifestyle:Zinc**	0	Some concerns	Low risk	No concerns	Major concerns	No concerns	Major concerns	Very low	Imprecision and Incoherence
		**Mixed:Omega 3**	0	Some concerns	Low risk	No concerns	Major concerns	No concerns	Major concerns	Very low	Imprecision and Incoherence
		**Mixed:Zinc**	0	Some concerns	Low risk	No concerns	Major concerns	No concerns	Major concerns	Very low	Imprecision and Incoherence
		**Omega 3:Protein**	0	Some concerns	Low risk	No concerns	Major concerns	No concerns	Major concerns	Very low	Imprecision and Incoherence
		**Omega 3:Zinc**	0	Some concerns	Low risk	No concerns	Major concerns	No concerns	Major concerns	Very low	Imprecision and Incoherence
		**Protein:Zinc**	0	Some concerns	Low risk	No concerns	Major concerns	No concerns	Major concerns	Very low	Imprecision and Incoherence
**Lymphocyte**	**Mixed**	**Beta-carotene:Control**	1	Some concerns	Low risk	No concerns	Major concerns	No concerns	Major concerns	Very low	Imprecision and Incoherence
		**Control:Lifestyle**	1	Some concerns	Low risk	No concerns	Major concerns	No concerns	Major concerns	Very low	Imprecision and Incoherence
		**Control:Mixed**	2	Some concerns	Low risk	No concerns	Major concerns	No concerns	Major concerns	Very low	Imprecision and Incoherence
		**Control:Zinc**	2	Some concerns	Low risk	No concerns	Major concerns	No concerns	Major concerns	Very low	Imprecision and Incoherence
		**Glutamine:Protein**	3	Some concerns	Low risk	No concerns	Major concerns	No concerns	Major concerns	Very low	Imprecision and Incoherence
		**Mixed:Protein**	1	Some concerns	Low risk	No concerns	Major concerns	No concerns	Major concerns	Very low	Imprecision and Incoherence
	**Indirect**	**Beta-carotene:Glutamine**	0	Some concerns	Low risk	No concerns	Major concerns	No concerns	Major concerns	Very low	Imprecision and Incoherence
		**Beta-carotene:Lifestyle**	0	Some concerns	Low risk	No concerns	Major concerns	No concerns	Major concerns	Very low	Imprecision and Incoherence
		**Beta-carotene:Mixed**	0	Some concerns	Low risk	No concerns	Major concerns	No concerns	Major concerns	Very low	Imprecision and Incoherence
		**Beta-carotene:Protein**	0	Some concerns	Low risk	No concerns	Major concerns	No concerns	Major concerns	Very low	Imprecision and Incoherence
		**Beta-carotene:Zinc**	0	Some concerns	Low risk	No concerns	Major concerns	No concerns	Major concerns	Very low	Imprecision and Incoherence
		**Control:Glutamine**	0	Some concerns	Low risk	No concerns	Major concerns	No concerns	Major concerns	Very low	Imprecision and Incoherence
		**Control:Protein**	0	Some concerns	Low risk	No concerns	Major concerns	No concerns	Major concerns	Very low	Imprecision and Incoherence
		**Glutamine:Lifestyle**	0	Some concerns	Low risk	No concerns	Major concerns	No concerns	Major concerns	Very low	Imprecision and Incoherence
		**Glutamine:Mixed**	0	Some concerns	Low risk	No concerns	Major concerns	No concerns	Major concerns	Very low	Imprecision and Incoherence
		**Glutamine:Zinc**	0	Some concerns	Low risk	No concerns	Major concerns	No concerns	Major concerns	Very low	Imprecision and Incoherence
		**Lifestyle:Mixed**	0	Some concerns	Low risk	No concerns	Major concerns	No concerns	Major concerns	Very low	Imprecision and Incoherence
		**Lifestyle:Protein**	0	Some concerns	Low risk	No concerns	Major concerns	No concerns	Major concerns	Very low	Imprecision and Incoherence
		**Lifestyle:Zinc**	0	Some concerns	Low risk	No concerns	Major concerns	No concerns	Major concerns	Very low	Imprecision and Incoherence
		**Mixed:Zinc**	0	Some concerns	Low risk	No concerns	Major concerns	No concerns	Major concerns	Very low	Imprecision and Incoherence
		**Protein:Zinc**	0	Some concerns	Low risk	No concerns	Major concerns	No concerns	Major concerns	Very low	Imprecision and Incoherence
**CD4/CD8**	**Mixed**	**Beta-carotene:Control**	1	Some concerns	Low risk	No concerns	Major concerns	No concerns	Major concerns	Very low	Imprecision and Incoherence
		**Control:Lifestyle**	1	Some concerns	Low risk	No concerns	Major concerns	No concerns	Major concerns	Very low	Imprecision and Incoherence
		**Control:Mixed**	3	Some concerns	Low risk	No concerns	No concerns	Major concerns	Major concerns	Very low	Heterogeneity and Incoherence
		**Control:Zinc**	2	Some concerns	Low risk	No concerns	Major concerns	No concerns	Major concerns	Very low	Imprecision and Incoherence
		**Mixed:Protein**	1	Major concerns	Low risk	No concerns	Major concerns	No concerns	Major concerns	Very low	Imprecision and Incoherence
	**Indirect**	**Beta-carotene:Lifestyle**	0	Some concerns	Low risk	No concerns	Major concerns	No concerns	Major concerns	Very low	Imprecision and Incoherence
		**Beta-carotene:Mixed**	0	Some concerns	Low risk	No concerns	No concerns	Major concerns	Major concerns	Very low	Heterogeneity and Incoherence
		**Beta-carotene:Protein**	0	Some concerns	Low risk	No concerns	Major concerns	No concerns	Major concerns	Very low	Imprecision and Incoherence
		**Beta-carotene:Zinc**	0	Some concerns	Low risk	No concerns	Major concerns	No concerns	Major concerns	Very low	Imprecision and Incoherence
		**Control:Protein**	0	Major concerns	Low risk	No concerns	Major concerns	No concerns	Major concerns	Very low	Imprecision and Incoherence
		**Lifestyle:Mixed**	0	Some concerns	Low risk	No concerns	No concerns	Major concerns	Major concerns	Very low	Heterogeneity and Incoherence
		**Lifestyle:Protein**	0	Some concerns	Low risk	No concerns	Major concerns	No concerns	Major concerns	Very low	Imprecision and Incoherence
		**Lifestyle:Zinc**	0	Some concerns	Low risk	No concerns	Major concerns	No concerns	Major concerns	Very low	Imprecision and Incoherence
		**Mixed:Zinc**	0	Some concerns	Low risk	No concerns	Major concerns	No concerns	Major concerns	Very low	Imprecision and Incoherence
		**Protein:Zinc**	0	Some concerns	Low risk	No concerns	Major concerns	No concerns	Major concerns	Low	Imprecision and Incoherence
**Neutrophil**	**Mixed**	**Control:Lifestyle**	1	Some concerns	Low risk	No concerns	None	Unclear	Unclear	Unclear	Unclear
		**Control:Zinc**	2	Some concerns	Low risk	No concerns	None	Unclear	Unclear	Unclear	Unclear
	**Indirect**	**Lifestyle:Zinc**	0	Some concerns	Low risk	No concerns	None	Unclear	Unclear	Unclear	Unclear

## Supplementary Materials

The following are available online at https://www.mdpi.com/article/10.3390/metabo12090868/s1, All the original data are provided in the Supplementary File.
